# Multiparametric MRI and Radiomics in Prostate Cancer: A Review of the Current Literature

**DOI:** 10.3390/diagnostics11101829

**Published:** 2021-10-03

**Authors:** Federico Midiri, Federica Vernuccio, Pierpaolo Purpura, Pierpaolo Alongi, Tommaso Vincenzo Bartolotta

**Affiliations:** 1 Section of Radiology—BiND, University Hospital “Paolo Giaccone”, 90127 Palermo, Italy; federicavernuccio@gmail.com (F.V.); tommasovincenzo.bartolotta@unipa.it (T.V.B.); 2 Department of Radiology, Fondazione Istituto “Giuseppe Giglio”, Ct.da Pietrapollastra, Via Pisciotto, Cefalù, 90015 Palermo, Italy; pierpaolopurpura@gmail.com; 3 Nuclear Medicine Unit, Fondazione Istituto “Giuseppe Giglio”, Ct.da Pietrapollastra, Via Pisciotto, Cefalù, 90015 Palermo, Italy; alongi.pierpaolo@gmail.com

**Keywords:** radiomics, magnetic resonance imaging, prostate, cancer, PI-RADS, Gleason score

## Abstract

Prostate cancer (PCa) represents the fourth most common cancer and the fifth leading cause of cancer death of men worldwide. Multiparametric MRI (mp-MRI) has high sensitivity and specificity in the detection of PCa, and it is currently the most widely used imaging technique for tumor localization and cancer staging. mp-MRI plays a key role in risk stratification of naïve patients, in active surveillance for low-risk patients, and in monitoring recurrence after definitive therapy. Radiomics is an emerging and promising tool which allows a quantitative tumor evaluation from radiological images via conversion of digital images into mineable high-dimensional data. The purpose of radiomics is to increase the features available to detect PCa, to avoid unnecessary biopsies, to define tumor aggressiveness, and to monitor post-treatment recurrence of PCa. The integration of radiomics data, including different imaging modalities (such as PET-CT) and other clinical and histopathological data, could improve the prediction of tumor aggressiveness as well as guide clinical decisions and patient management. The purpose of this review is to describe the current research applications of radiomics in PCa on MR images.

## 1. Introduction

**Figure 1:**
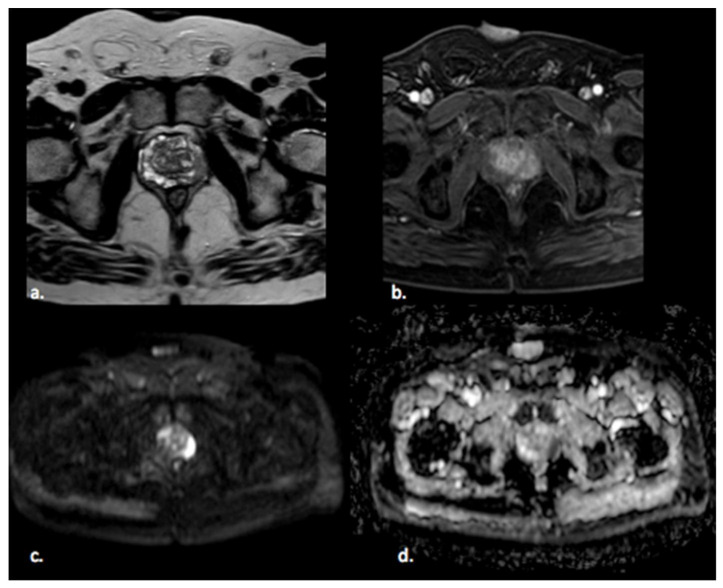
A 65 year old man with a PI-RADS 5 lesion in the left postero-lateral segment of the PZ of midgland, hypointense in T2-weighted images (**a**), with early enhancement in DCE images (**b**), markedly hyperintense on DWI, and hypointense on ADC images (**c**,**d**).

**Figure 2:**
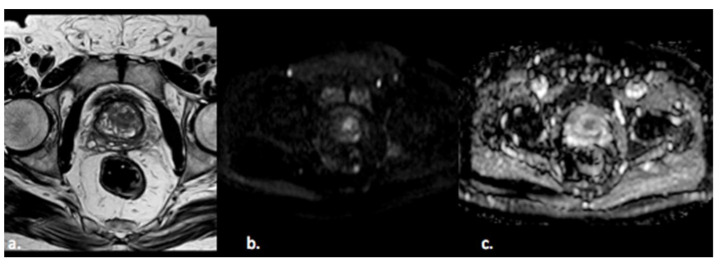
A 55-year old man with a PI-RADS 3 lesion in the left anterior segment of PZ of the midgland, moderately hypointense on T2-weighted images (**a**), hyperintense on DWI, and hypointense on ADC images (**b**,**c**).

Prostate cancer (PCa) is the fourth most commonly diagnosed cancer and the fifth leading cause of cancer death among men worldwide [1]. PCa more frequently (80%) originates in the peripheral zone (PZ) and less commonly (15%) in the transitional zone (TZ), while the central zone (CZ) location of PCa is rare [2]. Albeit less common, PCa in the TZ contributes to morbidity and mortality because of confounding changes in this region due to benign prostatic hyperplasia, which is found in up to 25% of TZ cancers [2]. Transrectal ultrasound (TRUS) is a cost-effective and easily available imaging modality, but with limited sensitivity and specificity ranging between 40% and 50% for detection of PCa [3]. Multiparametric MRI (mp-MRI) has gained popularity as a noninvasive imaging technique for detection of clinically significant PCa and biopsy guidance. mpMRI may overcome many of the shortcomings of the combination of PSA and TRUS alone, achieving accurate tumor detection with sensitivity of 72% and specificity of 81% [4,5,6]. It is also increasingly used in patients undergoing active surveillance to monitor recurrence in patients after radiotherapy (RT) or androgen deprivation therapy (ADT). The MRI diagnostic system for prostatic lesions is known as Prostate Imaging-Reporting and Data System (PI-RADS), and the latest version (v2.1) was published in 2019 [5]. This system evaluates the relative likelihood of the existence of a clinically significant prostate cancer ranging from PI-RADS 1 “clinically significant disease is highly unlikely to be present” to PI-RADS 5 “clinically significant cancer is highly likely to be present” (Figure 1). The PI-RADS scoring system has high sensitivity and specificity, but still there are many lesions that are categorized as PI-RADS 3 (Figure 2) or PI-RADS 4 which means that these lesions carry a moderate to high risk of being or becoming clinically significant prostate cancer but cannot be diagnosed as such, and biopsy may be needed [6,7].

In the last decade, there has been increasing interest in the quantitative analysis of imaging data. Radiomics is a relatively novel process of medicine designed to extract a large number of quantitative features from radiological images, offering a cost-effective and high-throughput approach to medical imaging data analysis using advanced mathematical algorithms, which could lead to accurate tumor detection and aid personalized cancer treatment [8,9]. Radiomics and artificial intelligence (AI) cover a wide variety of subfields and techniques. Machine learning is the subfield of AI where the algorithm is applied to a set of data and to knowledge about these data; radiologists can select and encode features that appear distinctive in the data, and the statistical techniques are used to organize the data on the basis of these features. Then, the system can learn from the training data and apply what it has learned to make a prediction (e.g., for differential diagnosis between benign or malignant lesions) [10]. Representation learning is a type of machine learning where the algorithm learns on its own the best features to classify the provided data. Deep learning is a type of representation learning where the algorithm learns a composition of features that reflect a hierarchy of structures in the data. This system is able to discriminate the compositional nature of images starting from simple features (intensity, edges, and textures) to elaborate more complex features such as shapes, lesions, or organs [11]. Thus, these systems are important in the use of radiomics in medical images because they allow collapsing clusters of big datasets into a few representative features and creating classifier models through database mining. In the last few years, deep learning has been applied to prostate cancer with promising results, although it is not yet used in the clinical routine.

The aim of this narrative review was to describe the current and potential radiomics applications for prostate cancer on mpMRI. For this purpose, we first describe the different steps of radiomic analysis, and then we provide a summary of the literature on radiomic analysis for prostate cancer.

## 2. Radiomics Analysis

**Figure 3:**
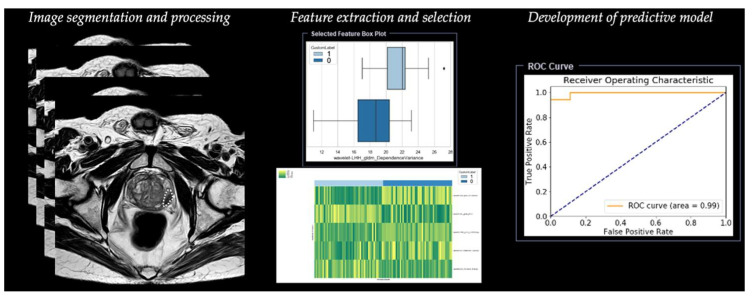
Workflow of radiomics for prostate cancer in a simulated study on T2-weighted images using a prototype research software Radiomics, version 1.0.9 (Siemens Healthineers, Forchheim, Germany).

Radiomic analysis requires different steps, including segmentation, image processing, feature extraction, feature development, and development of a predictive model (Table 1) (Figure 3).

### 2.1. Step 1—Segmentation

The first step is image segmentation of the region of interest (ROI) in two dimensions (2D) or of the volume of interest (VOI) in three dimensions (3D), defining the area in which radiomic features will be calculated. Image segmentation can be manual or semi-automatic (usually with manual correction), but this method is considered time-consuming and does not allow a reproducible analysis of the radiomic derived features for its intrinsic intra-observer variability [12]. Although there is still no universal segmentation algorithm for all image applications, the best option is automated image segmentation using atlas-based and model-based methods that avoid intra- and inter-observer variation [13]. These methods work well for relatively homogeneous lesions, but show the need for intensive user correction for inhomogeneous lesions, such as lesions including air voxels as one example. Haaburger et al. [14] proposed a neural network architecture that generates plausible segmentation after separate training using default parameters as provided in the reference implementation.

### 2.2. Step 2—Image Processing

The second step is image processing, and it represents the attempt to homogenize images with respect to pixel spacing, gray-level intensities, and bins of gray-level histogram. This step consists of interpolation to isotropic voxel spacing to increase reproducibility between different datasets, intensity outlier filtering (normalization) to remove pixels/voxels that fall outside of a specified range of gray-level, and discretization of image intensities, which consists of grouping the original values according to specific range intervals [12].

### 2.3. Step 3—Feature Extraction

The third step is the extraction of radiomic features. Since many different ways and formulas exist to calculate those features, adherence to the Image Biomarker Standardization Initiative (IBSI) is recommended [15].

Features extracted from diagnostic images are classified into two groups. The first group includes the so-called “semantic features”, represented by radiologic features commonly used to describe lesions such as shape, location, vascularity, and necrosis. The second group includes the so-called “agnostic features” that analyze lesion heterogeneity through quantitative descriptors which are subdivided in turn into first-, second-, or higher-order statistical outputs [8]. The distribution of individual voxel intensities without concern for a spatial relationship is described through first-order statistics. These features reduce an ROI to single values for mean, median, uniformity, or randomness (entropy), magnitude (energy), and minimum and maximum gray-level intensity. Second-order statistics, introduced in 1973 by Haralick [16], describe interrelationships between voxels with similar or dissimilar contrast values as “texture features”, and they can readily provide a measure of intratumoral heterogeneity; these features are based on the gray-level co-occurrence matrix (GLCM), defining the pattern of an image subregion by summarizing the appearance of voxel pairs with a specific discretized gray-level value in a specified direction, and on the gray-level run length matrix (GLRLM), summarizing the frequency of continuous voxels that have the same discretized gray-level value in a given direction [17]. Higher-order statistical methods impose filter grids to extract repetitive or nonrepetitive patterns [13].

### 2.4. Step 4—Feature Selection

The next step is represented by feature selection, performed to select the most useful subset of features to build statistical and machine learning models with the exclusion of nonreproducible, redundant, and nonrelevant features. Rizzo et al. [13] analyzed cluster analysis and principal component analysis, which are the two most commonly used unsupervised approaches. Cluster analysis creates groups of similar features (clusters), and a single feature may be selected from each cluster as representative and used in the following association analysis. Principal component analysis creates a smaller set of maximally uncorrelated variables from a large set of correlated variables, and it allows explaining the variation in the dataset with the fewest possible principal components. After the selection of the most representative features for each cluster, it is possible to develop a model fitting with these remaining features.

### 2.5. Step 5—Development of Predictive Model

Once features have been selected, they are used for training the predictive model. This is built with different machine learning algorithms, including support vector machine (SVM), logistic regression, random forest (RF), and decision tree (DT).

The rapid development of deep learning, such as convolutional neural network (CNN) and artificial neural network (ANN), has accelerated the pace of radiomics progress [18].

## 3. Radiomics in Prostate Cancer

In the last decade, radiomic studies have investigated the potential application of texture analysis for prostate tumor detection and diagnosis, as well as for the prediction of aggressiveness and treatment evaluation based on mpMRI findings (Table 2).

### 3.1. Detection of Prostate Cancer

The application of radiomics has improved the process of predictive model development for indicating tumor location, known as computer-aided detection (CAD). The intention of CAD systems is to advise and complement radiologists in PCa detection in both PZ and TZ, increasing the sensitivity from 74% to 100% and specificity in from 43% to 93% with a 1.5 Tesla MRI scanner, as reported by Lemaître et al. [34]. Deep learning networks for automated prostate segmentation through CAD using atlas-based and model-based methods could facilitate the radiomic analysis of the tissue and reduce the time needed for segmentation, as well as the operator variability [35]. In the last few years, many CAD systems have been developed to detect prostate cancer on MRI images. The first CAD system to detect PCa in the PZ was implemented by Chan et al. [19], using SVM as a classifier. In 2015, Giannini et al. [20] proposed a CAD system based on a two-stage process. Firstly, a parametric color-coded map of the prostate gland was created, and colors were assigned to the map as a function of the probability of each voxel being cancerous; then, a candidate PCa segmentation was performed to highlight suspected areas. This is a fully automated system, and it was trained using the histopathological images as a comparison to reduce the number of false positives after two steps of reduction.

### 3.2. Diagnosis of Prostate Cancer

Ginsburg et al. [2] evaluated features in a cross-institutional setting for cancer detection in TZ and PZ. Radiomic features for the identification of cancer detection in the PZ were distinct from those that were useful in the TZ [2]. 

In 2015, Wibmer et al. [21] investigated whether Haralick texture analysis (i.e., energy, entropy, homogeneity, and contrast from GLCM) could help in differentiating between clinically and non-clinically significant PCa on mp-MRI, with good results for the analysis of T2-weighted and ADC images. Cameron et al. [22] proposed a model consisting of an initial tumor candidate identification schema followed by the MAPS system (morphology, asymmetry, physiology, size) to score the candidate regions; the goal of the proposed model was to incorporate high-level features using candidate tumor regions through mp-MRI and region morphology to construct a high-dimensional feature space that can be mined for different purposes such as detection or prognosis of cancer, achieving an accuracy of 87%, sensitivity of 86%, and specificity of 88%. Moreover, Bleker [23] in 2020 realized a model based on mp-MRI features extracted from an auto-fixed volume of interest (VOI) that quantifies the phenotype of clinically significant PCa in PZ on the basis of T2W and DWI images; DCE features improved diagnostic performance, despite not being statistically significant.

Lastly, Khalvati et al. [24] designed a new automatic mp-MRI texture feature model incorporating computed high-b diffused weighted imaging and correlated diffusion imaging that improved the visual separability of cancerous and healthy tissue in the prostate, leading to improved performance in both detecting PCa and prognosis, achieving a sensitivity, specificity, and accuracy of 82%, 89%, and 86%, respectively.

### 3.3. Grading and Aggressiveness

Accurate assessment of localized prostate cancer aggressiveness is of utmost importance for determining patient treatment and follow-up strategies. The Gleason score (GS) is the current clinical grading system for prostatic carcinoma, which is based only on the architectural pattern of the tumor. The grade is defined as the sum of the grade of the two most common architectural alterations of the prostatic tissue [36]. The discrimination between clinically significant (GS equal or more than 7) and non-clinically significant PCa (GS equal to 6) is important to reduce unnecessary biopsies and to avoid unnecessary prostatectomy. Indeed, non-clinically significant lesions are often detected, and clinically significant cancers are sometimes missed. TRUS biopsy also carries significant morbidity, such as erectile dysfunction and urinary incontinence, and it can cause life-threatening sepsis [37]. Radiomic combined patterns can impact clinical outcomes, treatment selection, and the determination of disease status noninvasively, which avoids unnecessary invasive instrumentation. Many studies demonstrated how texture features, such as energy, entropy, contrast, and homogeneity on GLCM, calculated on T2W and ADC images, could provide information related to aggressiveness, differentiating low-risk from intermediate- or high-risk PCa [21,38,39,40,41].

In 2015, Fehr et al. [25] proposed a machine learning-based automatic classification (RFE-SVM, recursive feature selection support vector machine) of PCa aggressiveness by combining ADC and T2W images. This method distinguished between GS 6 (3 + 3) and ≥7 cancers with 93% accuracy; moreover, this approach distinguished GS 7 (3 + 4) from GS 7 (4 + 3) with 92% accuracy. In comparison, a classifier using only the ADC mean achieved an accuracy as high as 58% for distinguishing GS (3 + 3) from GS ≥ 7 and 59% for distinguishing GS 7 (3 + 4) from GS 7 (4 + 3).

Nketiah et al. [26] demonstrated that T2W image-derived textural features were correlated significantly with GS; in particular, homogeneity and entropy were significantly different between GS 7 (3 + 4) and GS 7 (4 + 3). Similar to Fehr [26], the results indicated that combining traditional MRI and derived textural features could achieve a higher classification accuracy (91%). Then, they tested and confirmed with a multicenter study the potential of T2W image-derived textural features for quantitative assessment of peripheral zone PCa aggressiveness. Statistical analysis with the Mann–Whitney U test indicated that image homogeneity and disorder/complexity correlated significantly (*p* < 0.05) with low- (grade group 1) and intermediate/high-risk (grade group ≥ 2) PCa [27].

The study of Cuocolo et al. [28] recognized the surface area-to-volume ratio (SAVR) derived from ADC maps as a promising tool in the discrimination of clinically and non-clinically significant PCa, outperforming other shape features (AUC = 0.78). Assessment of geometric parameters has the potential to be used as a noninvasive test to predict GS for patients with clinically significant PCa.

Chaddad et al. [29,42] proposed a model based on the joint intensity matrix (JIM) to predict the GS of PCa. JIM is a computation that translates image heterogeneity into texture predictors on the basis of five different derived features (contrast, homogeneity, difference variance, dissimilarity, and inverse difference), and it has the capacity to compare GS groups (GS 6, GS 3 + 4, and GS ≥ 4 + 3); higher-Gleason-score cancers have been found to be associated with relatively high ADC entropy and low ADC energy, in comparison with low-Gleason-score cancers [29,42].

### 3.4. Radiomics and PI-RADS Score

The interpretation of PI-RADS 3 lesions remains often undefined, termed as “intermediate” or “equivocal in the presence of clinically significant cancer” [5]. These lesions represent a treatment challenge for urologists, ranging from conservative management with imaging follow-up to surgical therapy. Some studies investigated the role of machine learning-based classifiers in detecting clinically significant PCa with PI-RADS score 3 lesions. Results predicted by the classifier may be an important reference for clinical decision making and will help in increasing the prostate-positive biopsy rate in PI-RADS 3 while decreasing unnecessary biopsies [43,44,45]. Giambelluca et al. [30] showed that predictive models based on texture features extracted through a texture analysis software (MaZda 4.6) had a good performance for the diagnosis of clinically significant PCa among PI-RADS 3 lesions on T2W (AUROC = 0.77) and ADC map (AUROC = 0.81) images. These results may provide preliminary evidence to justify the use of texture analysis in the stratification of PI-RADS 3 lesions.

Another limitation of PI-RADS 3 lesion assignment concerns the role of DCE-MRI, which is still debated and not clearly assessed. Currently, DCE-MRI is only used to upgrade PI-RADS category 3 lesions to PI-RADS category 4, but only for lesions located in the PZ [5]. Brancato et al. [31] examined the mp-MRI-based radiomic approach and was able to improve PI-RADS v2.1 performance in stratifying PI-RADS 3 and PI-RADS 4 lesions. Lesions with Gleason score ≥6 on biopsy were used as the reference standard. The most relevant features for classification were texture features arising from T2W and ADC images; the features associated with DCE-MRI were not useful for building a predictive model.

Figure 3 depicts an example of the workflow of radiomics for prostate cancer [46].

### 3.5. Treatment Evaluation and Prediction of Biochemical Recurrence

Prostate cancer patients are typically classified into different categories as a function of the PSA level, Gleason Score, and T stage (tumor size) as low- (PSA ≤ 10 ng/mL, GS ≤ 6, T1–T2), intermediate- (10 ng/mL < PSA < 20 ng/mL, GS = 7, T2b), and high-risk (PSA > 20 ng/mL, GS ≥ 8, T2c–T3a) [47]. In clinical practice, patients who undergo whole-gland radiotherapy (RT) may have short-term and long-term side effects such as incontinence, sexual dysfunction, and bowel toxicity. Abdollahi et al. [48] demonstrated the application of radiomic features to assess radiation-induced bladder wall changes and the relationship that exists between radiation dose and change in these features, using pre- and post-radiotherapy images. The most significant feature change occurred in the GLCM feature set; radiation breaks tissue homogeneity, and the radiomic changes are correlated with radiation dose.

In local therapy, it is important to localize malignant lesions accurately to increase biological effects against the tumor while achieving a reduction in target dose to noncancerous tissue. For this purpose, Shiradkar et al. [32] presented a radiomics-assisted targeted treatment radiotherapy planning (Rad-TRaP), consisting of three modules: cancer detection on MRI based on radiomic feature analysis, transference of cancer delineation to CT via multimodal deformable co-registration, and then generation of targeted focal radiotherapy plans for brachytherapy and external beam radiation therapy (EBRT).

Biochemical recurrence (BCR) is one of the most frequent complications of patients undergoing radical prostatectomy, especially those categorized as high-risk cancer (PSA > 20 ng/mL, GS ≥ 8, T2c-T3a) [49]. Some studies explored the association between extracted textural features from MR images and biochemical recurrence. Gnep et al. [33] showed how geometrical characteristics related to the tumor size and Haralick texture features (inverse difference moment, sum of squares, and difference entropy), derived primarily from T2W images, correlate with Gleason score and are associated with biochemical recurrence.

## 4. Limitations and Future Applications

Despite the wide range of potential applications, radiomics may be sensitive to a number of technical factors. First of all, the main challenge is the lack of reproducibility and uniform standardization in acquisition protocol and feature selection. Most of the existing studies are retrospective with relatively small sample sizes, for which conclusions are short of extensive validation. Moreover, the number of extracted features is much greater than the number of patients, which can lead to feature selection bias [50].

The future directions include the correlation between proteomic and genomic tumor analysis with radiomic features through the introduction of radiogenomics. Currently, the application of this technique in PCa is relatively less diffused than in other organs such as brain or lung. This new radiomic approach will play an important role in the future directions of personalized treatment in patients affected by PCa in order to increase the diagnostic performance of imaging mp-MRI in predicting prognosis and treatment response. Characterization of the protein profile could reveal significant differences between benign tissue and tumors, as well as between low- and high-grade tumors. As reported by Skvortsov et al. [51], significant upregulation of HSP60 is shown in early and advanced PCa compared to nonmalignant tissue, while levels of lamin A expression are correlated with lower and higher Gleason scores. A multimodal approach combining different imaging modalities, such as CT, MRI, and PET, and including clinical and laboratory information could represent the future approach of an integrated radiomics model for PCa patients. PET/CT radiomics could provide promising pretreatment and intra-treatment biomarkers for outcome prediction, improving the limitation of each single technique to predict tumor prognosis [52].

## 5. Conclusions

In conclusion, radiomics has the potential to become a useful reproducible assistant tool in clinical oncology imaging, contributing to the main future objective of personalized diagnosis and treatment of prostate cancer patients.

## Figures and Tables

**Table 1:** Summary of main steps for radiomics analysis.

Step Number	Type of Process	Description of the Step
1	Segmentation	Manual, automatic, or semiautomatic segmentation of the images to define the region or volume of interest
2	Image processing	Processing of images to increase reproducibility
3	Feature extraction	Feature descriptors are used to quantify characteristics of the gray levels within the region or volume of interest
4	Feature selection	Selection of the most useful features and exclusion of nonreproducible features to create a statistical model
5	Development of predictive model	Development of a classifier with different machine learning algorithms

**Table 2:** Summary of most relevant studies on radiomic studies for prostate cancer.

Type of Study	Authors, Year	Description
Detection of PCa	Chan et al., 2003 [19]	Development of one of the first predictive models using support vector machine (AUC 0.71–0.80)
	Giannini et al., 2015 [20]	Parametric color-coded map of the prostate based on the probability of each voxel to be tumoral (AUC 0.83–0.98)
Diagnosis of PCa	Ginsburg et al., 2017 [2]	Evaluation of different radiomic features in PZ and TZ tumors (AUC 0.61–0.71)
	Wibmer et al., 2015 [21]	Haralick texture analysis to differentiate clinically significant and not clinically significant PCa
	Cameron et al., 2016 [22]	Development of a comprehensive feature model consisting of an initial tumor candidate identification schema (AUC 0.81–0.93)
	Bleker et al., 2020 [23]	Development of a model that quantifies the phenotype of clinically significant PCa in PZ based on T2W and DWI images (AUC 0.75–0.98)
	Khalvati et al., 2015 [24]	New automatic texture feature models incorporating computed high-b diffused weighted imaging (CHB-DWI; AUC 0.73–0.85) and correlated diffusion imaging (CDI; AUC 0.81–0.90) to improve differentiation of tumoral and healthy tissue
Grading and aggresiveness	Fehr et al., 2015 [25]	Development of an automatic classification with a high accuracy combining ADC and T2W to evaluate the aggressiveness of PCa (AUC 0.93)
	Nketiah et al., 2017; 2021 [26,27]	Texture features, such as homogeneity and entropy, could reveal the aggressiveness of peripheral PCa distinguishing GS 7 (3 + 4) and GS 7 (4 + 3) (AUC 0.83 vs. 0.72 of MRI parameters)
	Cuocolo et al., 2019 [28]	Geometric parameters, such as surface area-to-volume ratio (SAVR), could predict clinically and non-clinically significant PCa (AUC 0.78)
	Chaddad et al., 2018 [29]	Model based on the joint intensity matrix (JIM) to predict the GS through different derived-features and comparing between GS groups: GS 6 (AUC 0.83), GS 3 + 4 (AUC 0.72), and GS ≥ 4 + 3 (AUC 0.77)
PI-RADS score	Giambelluca et al., 2021 [30]	Development of a texture analysis model to diagnose clinically significant PCa withing PI-RADS 3 lesions larger than 5 mm on T2W (AUC = 0.77) and ADC map (AUC = 0.81) images.
	Brancato et al., 2021 [31]	Relevant texture features for stratifying PI-RADS 3 (AUC 0.80) and PI-RADS 4 lesions (AUC 0.89) from T2W and ADC images
Treatment evaluation and prediction of biochemical recurrence	Shiradkar et al., 2016 [32]	Targeted treatment radiotherapy planning based on a radiomic model, consisting of cancer detection on feature analysis, transference of delineation to CT, and generation of targeted focal radiotherapy plans
	Gnep et al., 2017 [33]	Geometrical characteristics and Haralick texture correlate with Gleason score, and they are associated with biochemical recurrence

## Data Availability

Not applicable.
